# Nanolignin–polypyrrole nanocomposites: synthesis, electrical properties, and their performance in energy conversion applications

**DOI:** 10.1039/d5ra04454e

**Published:** 2025-08-18

**Authors:** Ebtesam H. A. Eladgham, Mona H. Abdel Rehim, Gamal Turky

**Affiliations:** a Solar Energy Department, Engineering Research Institute and New and Renewable Energy, National Research Centre 33 El Behooth St., Dokki Giza Egypt; b Packing and Packaging Materials Department, Institute of Chemical Industries Research, National Research Centre 33 El Behouth St., Dokki Giza Egypt monaabdelrehim23@gmail.com; c Microwave Physics and Dielectrics Department, Physics Research Institute, National Research Centre 33 El Behooth St., Dokki Giza Egypt

## Abstract

Nanocomposites based on nanolignin (NL) and polypyrrole (PPy) are prepared *via in situ* oxidation polymerization. The chemical structure of the obtained nanocomposites was studied using FTIR and XRD and their morphology was studied by SEM. Broadband dielectric spectroscopy (BDS) was used to study the electrical and dielectric properties of the nanocomposites. The results showed that the dc-conductivity decreases remarkably as the NL concentration increases. It decreases significantly from 2.88 × 10^−5^ to 1.82 × 10^−8^ S cm^−1^ as the NL concentration increases tenfold in the 5% composite form. However, an interfacial polarization that originated from the accumulation of charge carriers at PPy–NL interface became clearer as NL concentration increased. Moreover, NL exhibits weak electrical conductivity due to its semiconducting nature, resulting in a band gap of 1.25 eV in the near-infrared (IR) region. Pure NL yields low efficiencies when used as an electrolyte in dye-sensitized solar cells (DSSCs). However, incorporating the conducting polymer PPy significantly improves the performance of NL-based electrolytes. Increasing the NL ratio to 50% optimized the DSSC efficiency, leading to enhanced performance. Overall, the study demonstrates that the integration of PPy with NL significantly promotes the electrical and dielectric properties of the nanocomposites, improving their performance in energy conversion applications.

## Abstract 2

A novel class of NL-PPy nanocomposites is synthesized *via* an *in situ* oxidative polymerization method. The electrical properties of the material and its performance as a redox-active electrolyte in dye-sensitized solar cells were studied.

## Graphical abstract

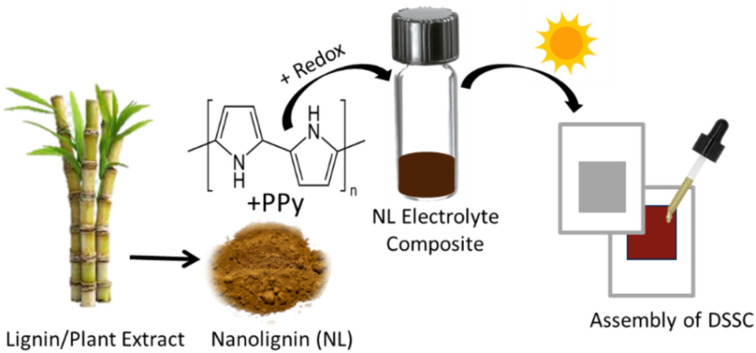

## 1. Introduction

Lignin is an agricultural bio-waste that represents nearly 30% of the cell walls in woody plants. It is a three-dimensional macromolecule considered the most abundant naturally occurring polymer on earth after cellulose.^1^ Lignin is an aromatic polymer of a chemical structure composed of several active subunits such as *p*-coumaryl alcohol, sinapyl alcohol, and coniferyl alcohol.^2^ The presence of a large amount of functional groups facilitates lignin chemical functionalization and utilization as lignin-based materials to obtain new biopolymers.^3^ Etherification,^4,5^ esterification,^6^ urethanization,^7,8^ and phenolation^9,10^ were described as possible chemical methods for lignin modification. On the way to added-value materials, the compositing of lignin with different polymers was described.^11^ Decreasing the size of lignin particles to the nanoscale has received high interest because of the newly improved properties of the nanolignin (NL) which opens the way to novel applications.^12^ Synthesis of NL from different lignin natural resources using chemical or physical methods was described.^13–18^ Moreover, the formation of NL/polymer hybrids extends their applications to broader limits such as UV blocking, ecofriendly packaging,^19,20^ sensors and energy.^21,22^

The use of lignin in optoelectronic applications, including light-emitting diodes and various types of photovoltaic devices (such as polymer, organic, and dye-sensitized solar cells), has been reported.^23^ Efforts are being made to incorporate lignin into different components of photovoltaic cells, where it can serve multiple functions, to improve the overall stability and efficiency of the device. Reports on lignin extracted from various sources and combined with TiO_2_ photoanodes have focused on improving the mechanical stability and binding of semiconductor oxides.^24–28^ It was found that addition of 5% of lignin extracted from rice husk to the TiO_2_ optimized the efficiency to 4.81%.^25^ The color of the extracted lignin is critical in such applications as dark-colored is inactive in the UV-visible region.^29^ Gianmarco Griffini *et al.* utilized a lignin membrane electrolyte in DSSCs with a polymer matrix, achieving the highest efficiency (∼1.5%) at a lignin/polymer mass ratio of 1.^30^ Functional groups in lignin such as hydroxyl, carboxyl, and carbonyl groups interact with the polymer or the semiconductors during the formation of the composite.^31,32^ Grafted sulfonated acetone–formaldehyde lignin (GSL) features a long-conjugated chain with hydroxyl groups, enhancing its hole-transporting properties with a reported overall efficiency of 14.49%.^33^ GSL was stabilized and doped with PEDOT to improve film homogeneity and enhance efficiency.^23^ Lignosulfonate (SL) and its cross-linked polymer with an alkyl carbon chain (ASL) were used as hole-transporting materials (HTMs).^34^ SL exhibited superior efficiency compared to its corresponding polymer, which was attributed to the essential presence of phenolic groups in the hole-transporting material. These groups are diminished upon polymerization and interaction with the alkyl chains, reducing their effectiveness. SL and ASL as well were also used as dopants in PEDOT, leveraging their aggregation properties and high hole mobility.^35^

Conductive polymers are a class of macromolecules distinguished by chemical structure of conjugated double bonds. Since they show properties of polymers and metals, they were called “synthetic metals”.^36^ They exhibit excellent electrical conductivity and ion mobility making them ideal for enhancing the performance of electrolyte systems in organic photovoltaics.^37^ Conductive polymers include polyaniline, PPy, and polythiophene. The most extensively studied polymer among them is PPy due to its biocompatibility, good thermal and environmental stability and high electrical conductivity.^38,39^ PPy can be prepared either by electrochemical or chemical oxidative polymerization techniques. The chemical oxidative polymerization method is the most widely used due to its simplicity and efficiency, offering a rapid route to obtain the polymer with a high yield.^40^ The polymerization reaction can be carried out in an organic solvent or aqueous medium at low pH value in the presence of a suitable oxidizing agent. Synthesis of PPy in an aqueous media is the most attractive route for researchers.^41^ The polymerization reaction mechanism begins with the coupling of two pyrrole radical cations, which are generated through the oxidation of the monomer by the oxidant during the initiation step.^42^ The resulting bipyrrole, after deprotonation, is subsequently oxidized and undergoes further coupling with another bipyrrole. Repeated oxidation, coupling, and deprotonation steps result in the formation of soluble segments, ultimately leading to the production of PPy as a black, insoluble powder. Admixing conductive PPy with naturally extracted NL shows great potential for a wide range of applications. Carbon- and nitrogen-rich composites were synthesized from PPy/lignin through pyrolysis in a nitrogen atmosphere at 650 °C. The material was produced in different media (alkaline, acidic, and neutral), and all were examined for their capacitive properties.^43^ Additionally, Hur *et al.* reported a PPy–lignin nanocomposite for use as an electrical sensor and in the removal of toxic metal ions.^44^

The BDS is established in terms of the large body of literature on the dielectric feature of the dipolar materials and moderately conducting electrolyte systems. BDS studies the relationship between both complex parameters (permittivity ε*(*ω*), and conductivity and σ*(*ω*)), on one hand, *via* phenomenological and molecular theories, the empirical relaxation functions and time-dependent molecular properties on the other hand give information on the rotational and translational motions of molecules and charges in molecular-liquids and solids.

This manuscript reports on the development of a novel class of nanolignin–polypyrrole nanocomposites, synthesized *via* an *in situ* oxidative polymerization method. The core novelty resides in the utilization of nanolignin as both a structural scaffold and a dopant-like component, which significantly influences the morphology and electrical properties of the resulting nanocomposite. We systematically investigate the electrical conductivity of these materials and, for the first time, demonstrate their successful application as a redox-active electrolyte in dye-sensitized solar cells. To achieve our goal, composites of polypyrrole (PPy) and NL in varying ratios were prepared using the *in situ* polymerization method. The chemical structures of the obtained materials were analyzed using various techniques, and their morphology was examined *via* SEM. The electrical properties of the nanocomposites were further investigated using BDS. DSSCs were fabricated by incorporating PPy/NL composites in different ratios as the electrolyte. This study aims to evaluate how the inclusion of NL influences the overall efficiency of the DSSCs.

## 2. Materials and methods

### 2.1 Materials

Soda lignin extracted from Bagasse was prepared in the labs of paper and cellulose department – National Research Centre – Egypt. The monomer, pyrrole (98%) is a product of Alfa Asser. Potassium persulphate (PPS), potassium bromide (KBr), tetrahydrofurane (99.9%), propylene carbonate (PC), ethylene carbonate (EC) anhydrous 99%, and 1-methyl-3-propylimidazolium iodide (IMII) are obtained from Sigma-Aldrich. Indium tin oxide (ITO) conductive glass is purchased from KINTEC.HK, China. Titanium dioxide (TiO_2_) P25 is a product of Degussa, Germany. N3 or Ru-535, and platisol solution (transparent platinum catalyst paint), were purchased from Solaronix, Switzerland. Triton® X-100 for gas chromatography is obtained from Merck. Polyethylene oxide 20 000 (PEO 20 000), acetylacetone 99.3% were purchased from Fluka. Polycarbonate sheet is obtained from General Electric, USA. Acetonitrile (AN) and iodine (I_2_) pearls are from PA-ACS, PANREAC Quimica SA. Potassium iodide (KI) extra pure are products of SD Fine-Chem Ltd, Mumbai. Barium sulphate (BaSO_4_), acetone, isopropanol extra pure AR and ethyl alcohol are products of Adwic. All chemicals are used without further treatment.

### 2.2 Methods

#### 2.2.1 Synthesis of PPy

10.0 g of the oxidant PPS is dissolved in 100 mL cold distilled water then pyrrole monomer (2.23 g, 0.03 mol) is added drop by drop. A black precipitate is formed immediately and the reaction was left under stirring for 24 hours. The formed polymer is separated by filtration, washed and dried in a vacuum oven at 70 °C.

#### 2.2.2 Synthesis of NL

2 g of lignin is dissolved in 200 mL of THF and stirred with a magnetic stirrer till complete dissolution. 160 mL of Millipore water, as non-solvent, is added in a rate of 4 mL min^−1^. Finally, THF is removed by rotary evaporator and a colloid of NL particles is obtained.

#### 2.2.3 Synthesis of PPy/NL

Preparation of the nanocomposite was performed as follows: 10.0 g of the oxidant PPS is dissolved in 40 mL cold distilled water then different amounts NL colloid namely (5, 10, 20 and 50 mL) were added to obtain nanocomposites of different concentrations. The overall volume of the polymerization solution was kept 100 mL. Amount of monomer is added and the reaction proceeded for 24 hours. The formed nanocomposite is separated by filtration, washed several times and finally dried.

#### 2.2.4 Fabrication of DSSC

**Fig. 1:**
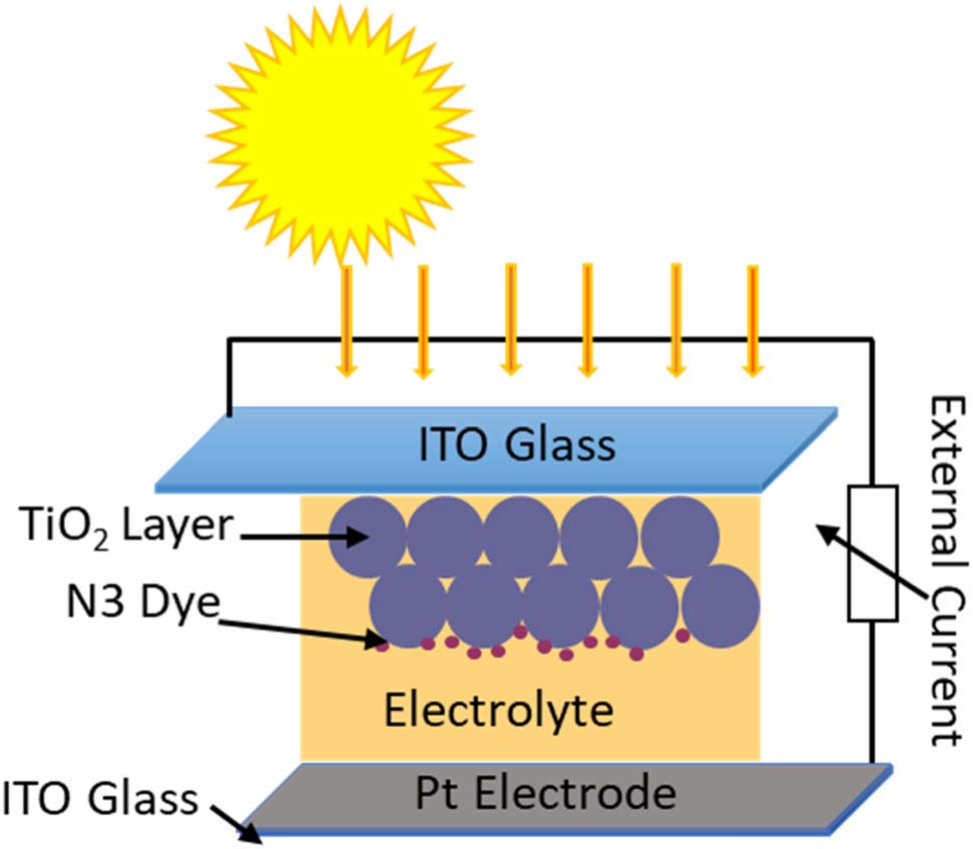
Schematic diagram of DSSC fabrication.

The fabrication of the DSSCs was discussed in detail in our previous publication.^37^ Briefly, TiO_2_ photo- and Pt counter electrodes were sandwiched against each other with the electrolyte in between as illustrated in Fig. 1.

##### 2.2.4.1 Photoelectrode

A 0.5 × 0.5 cm^2^ active area of ITO conductive glass was defined using 35 μm-thick tape to enable uniform deposition of the TiO_2_ film *via* the doctor blade technique, using 1–2 drops of prepared paste. The paste was prepared by grinding 6 g of TiO_2_ anatase (particle size < 25 nm) with 33 wt% PEO (*M*_w_ 20 000), 14 mL of deionized water, and 0.2 mL of acetylacetone for 30 minutes. Subsequently, 2 drops of Triton X-100 were added, followed by an additional 15 minutes of grinding. After deposition, the film was air-dried, the masking tape removed, and the substrate sintered in air at 450 °C for 30 minutes, then cooled to room temperature. The TiO_2_ films were subsequently immersed in a 5 × 10^−5^ mol L^−1^ N3 (Ru-535) dye solution in isopropanol after cooling to 80–60 °C and were sensitized overnight.

##### 2.2.4.2 Counter electrode

Two to three drops of platisol solution (transparent platinum catalyst paint) were evenly spread onto a clean ITO substrate using a glass rod. The solvent was allowed to evaporate at room temperature over a few minutes. The coated substrates were then fired in air at 450 °C for 15–30 minutes, resulting in the formation of small clusters of catalytically active platinum.

##### 2.2.4.3 The electrolyte

The electrolyte was prepared by dissolving 0.01 g of PPy/NL composite was dissolved in AN in a sealed glass vial with continuous stirring at 70 °C for 1 h. In another vial, 0.5 M KI and 0.5 M I_2_ were dissolved in a (9 : 1 vol) PC/EG mix. The iodine mixture is then transferred to the polymer vial and 0.5 M IMII was added and stirred for 1 h before raising the temperature to 90 °C for 24 h. Liquid electrolyte: 0.5 M KI and 0.5 M I_2_ were dissolved in a (9 : 1 vol) EG/AN mix.

##### 2.2.4.4 Cell assembly

A 50 μm polycarbonate spacer sheet was cut to match the cell borders and used to separate the two electrodes, effectively preventing short-circuiting. After electrolyte injection, the cell was sealed by clamping two opposite sides with paper clips to minimize electrolyte leakage.

### 2.3 Characterization

FTIR spectroscopy was used to investigate the chemical structure of the prepared. An instrument from Bruker VERTEX 80 ATR-FTIR (Germany) combined with Platinum Diamond ATR, comprising a diamond disk, was used. The instrument has internal reflector measure spectra in the range of 400–4000 cm^−1^ with a resolution of 4 cm^−1^ and a refractive index of 2.4. The structure of prepared samples was further studied using powder X-ray diffraction (PXRD) technique and a diffractometer from Empyrean Panalytical Instruments in the Netherlands, with CuK as an X-ray source of radiation. Scanning electron microscope (SEM), TESCAN VEGA3 was utilized to study sample morphology. UV spectrometer (Shimadzu, Germany) equipped with an internal diffuse reflectance accessory (DRA) is used to study optical properties. The powder samples were dispersed in a non-absorbing medium (BaSO_4_), so that scattering is minimized.

The performance of the DSSCs was investigated using a 300 W ABA SCIENCETECH solar simulator (Canada) equipped with a xenon lamp and the *I*–*V* data was recorded and analyzed using a 20 W-2401 SOURCEMETER Keithley. Photo-intensity was fixed to 1 sun or 100 mW cm^−2^ and the equipment was calibrated using a verified reference silicon cell.

The dielectric characteristics of the samples are examined using a Novocontrol high-resolution alpha analyzer in the frequency range of 10^−1^ to 10^7^ Hz. This method uses pure nitrogen as the heating agent and is accompanied by a Novocontrol-Quatro temperature controller system. Even at low volume fractions, dielectric spectroscopy is an effective technique for identifying and examining the dynamics of polar segments that relax near nanoparticles. With a measurement uncertainty of less than ±0.5%, the measurements were made at various temperatures between 25 and 150 °C isothermally and temperature is controlled by the Quatro-Novocontrol cryo system and repeated three times. Two stainless steel plate electrodes, the upper one with a 20 mm diameter, and the lower one with a 40 mm diameter, were positioned in parallel geometry between the pristine PES and PES/NL film samples. The complex dielectric function ε*(*υ*) = *ε*′(*υ*) − i*ε*′′(*υ*), the dissipation factor: 
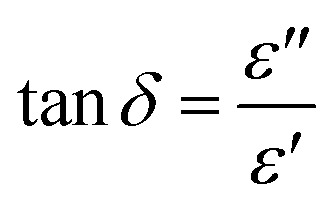
 (where, *ε*′ is the real part and *ε*′′ is the imaginary part or dielectric loss, 
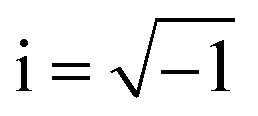
, *υ* is the frequency) were obtained.

## 3. Results and discussion

### 3.1 FTIR

**Fig. 2:**
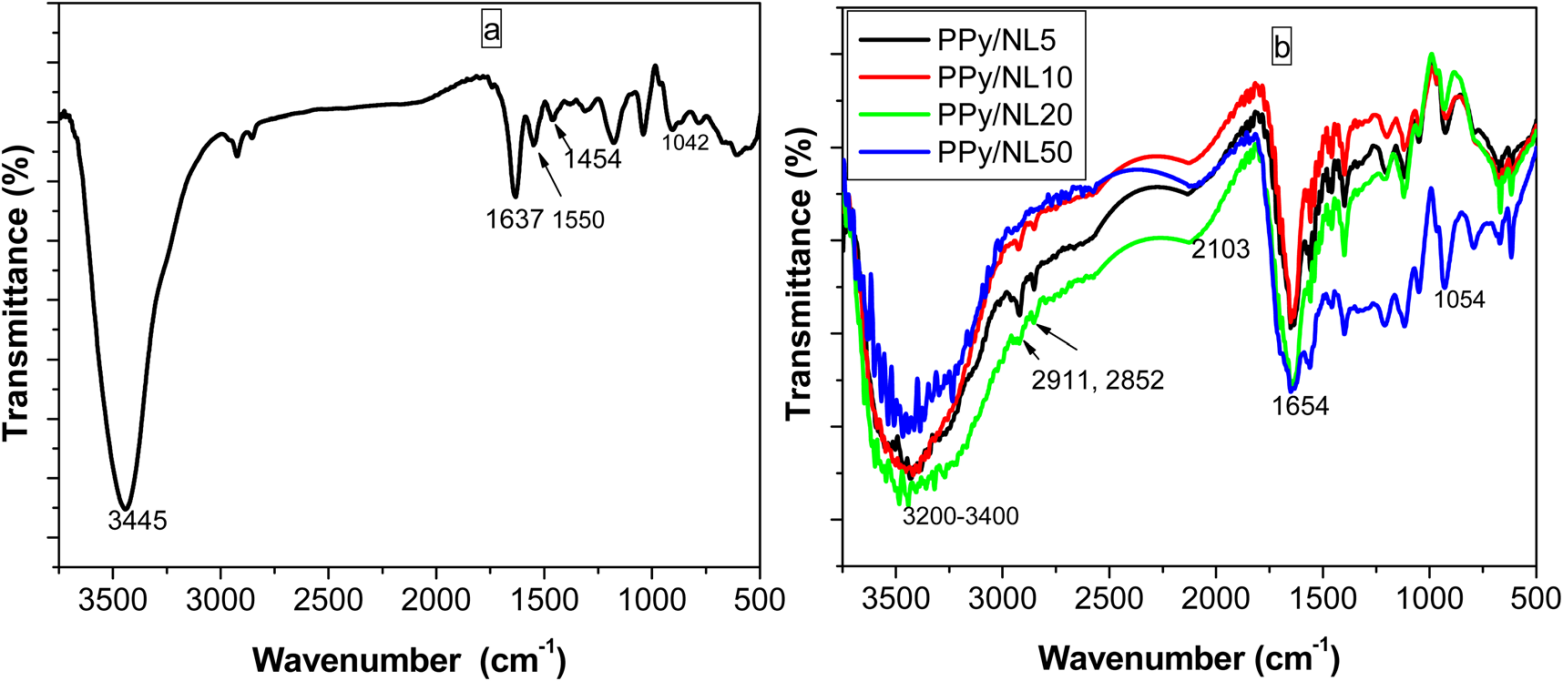
FTIR spectra of: [a] PPy, and [b] PPy/NL composites.

**Scheme 1:**
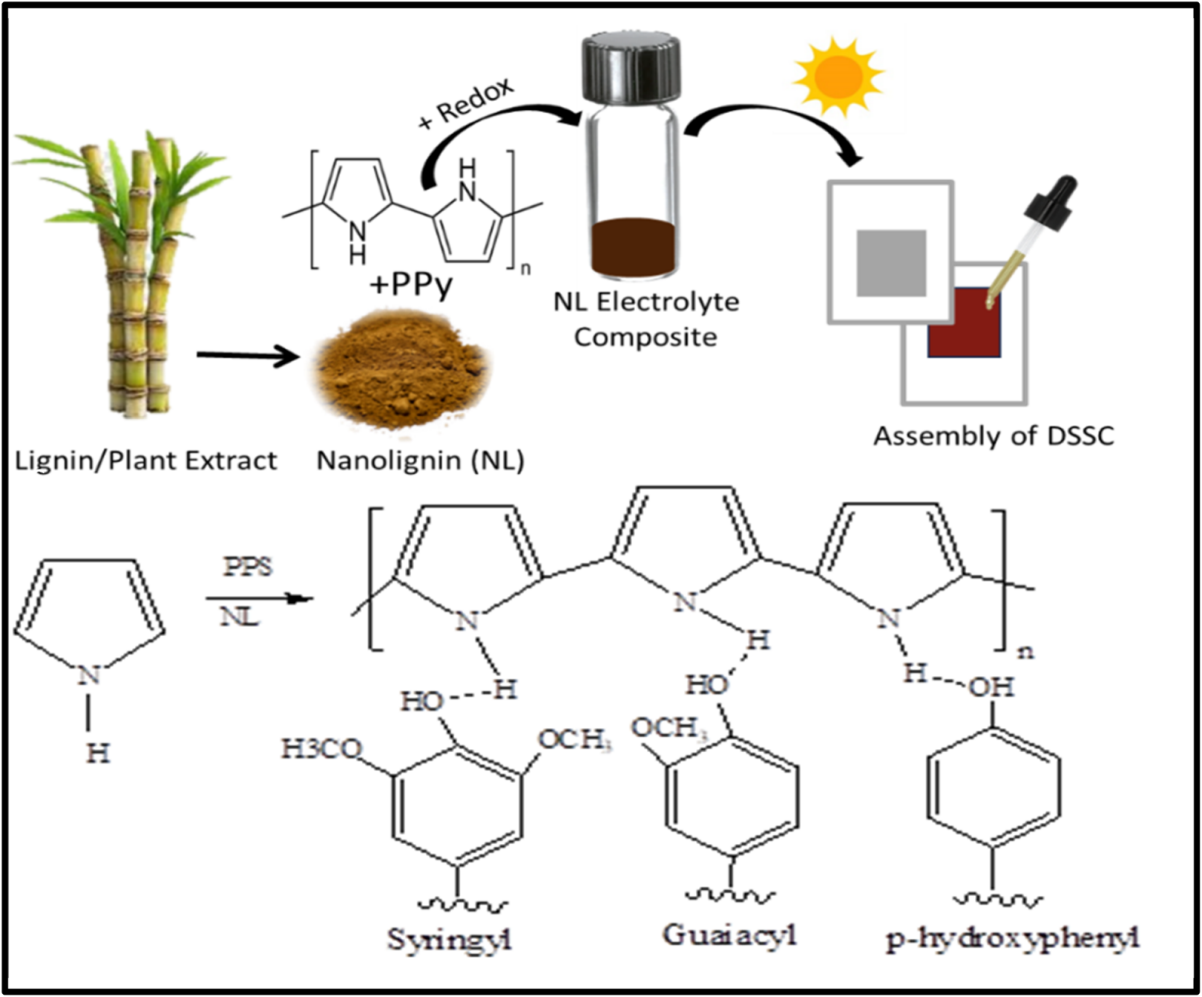
Synthetic steps of PP/NL composites, fabrication of the DSSC, and possible hydrogen bonds between PPy and different subunits in NL structure.

Chemical structures of PPy/NL composites compared to pure PPy were investigated by FTIR (Fig. 2) using KBr pellets.^45^ The spectrum of PPy depicted in Fig. 2a shows a band at 3445 cm^−1^ related to NH stretching vibration. The band at 1637 cm^−1^ can be attributed to C=O bond that appears in PPy prepared by PPS as oxidizing agent due to over-oxidation.^46^ The band at 1042 cm^−1^ is corresponding to =C–H in-plane deformation vibration.^47^ Bands corresponding to the vibration of the pyrrole ring and C=C appeared at 1454 and 1550 cm^−1^; respectively. However, Fig. 2b reveals FTIR spectra of different concentrations of PPy/NL hybrids. The spectra depict a broad band at 3200–3400 cm^−1^ is related to N–H and OH stretching vibration bands. Bands at 2911 and 2852 cm^−1^ are corresponding to aliphatic C–H. The weak band at 2103 cm^−1^ is due to C–H stretching vibration,^48^ while C=O band can be observed at 1654 cm^−1^. The =C–H in-plane vibration band is shifted to 1054 cm^−1^. Nevertheless, the shift in band positions in the prepared composites compared to PPy confirms the electrostatic interaction between PPy and nanolignin as shown in Scheme 1.

### 3.2 XRD

**Fig. 3:**
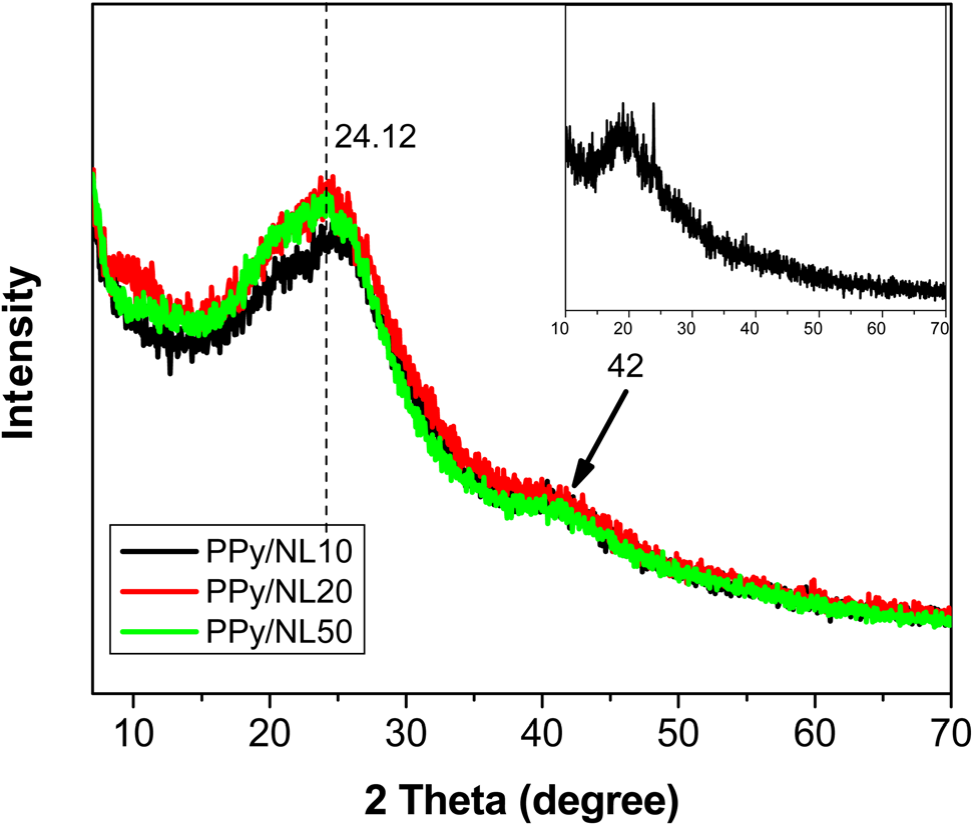
XRD pattern of polypyrrole/nanolignin composites compared with pure PPy (inset).

XRD diffraction patterns of PPy/NL composites compared to PPy (inset) are presented in Fig. 3. The XRD pattern of PPy showed one peak can be observed at 2*θ* = 20° related to the (002) plane and consistent with polymer chain diffraction confirming the amorphous structure of the polymer.^49^ Moreover, XRD investigations for PP/NL composites revealed 2 peaks, the main one shifted to 2*θ* = 24.12° and a small bump at 2*θ* = 42°. These findings suggest the formation of chain ordering in the obtained composite most likely due to the hydrogen bonds between PPy and NL functionalities as previously mentioned.

### 3.3 SEM

**Fig. 4:**
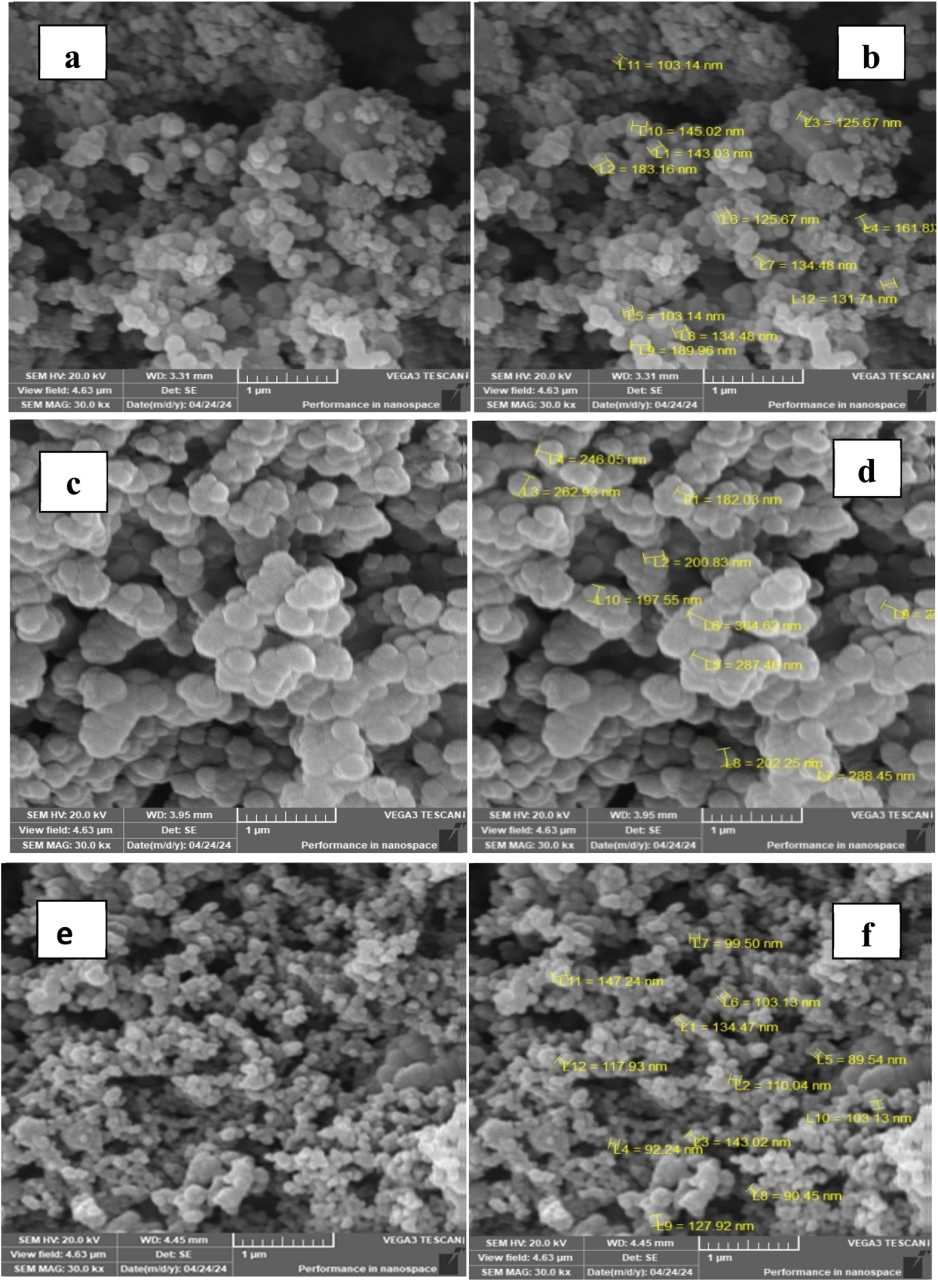
SEM images of [a and b] PPy, [c and d] PPy/NL5, and [e and f] PPy/NL50.

The morphology of PPy, PPy/NL5, and PPy/NL50 was investigated by scanning electron microscope, and the images are presented in Fig. 3. Images of PPy [Fig. 4a and b] revealed that the particles have spherical morphology similar to that observed before for PPy prepared by oxidation polymerization method.^50^ The particle size ranged from 103–189 nm (Fig. 4b). It should be pointed out that many factors affect particle size of the polymer such as temperature, and solvent concentration of the oxidant.^51,52^ The addition of 5% NL to the polymerization solution yielded polymer particles of irregular structure. The particle size increased remarkably with lowest particle diameter of 182 nm and reached 364 nm (Fig. 4d). It can be claimed that NL the addition of 5% of NL promoted larger particle size by acting as solid-state nuclei that adsorbed the formed oligomers and hence facilitated the evolution of the nanocomposite particles. Further increase in the concentration of NL in the polymerization medium led to the formation of nanocomposite of granular porous structure as depicted in Fig. 4e. The particle size shown in Fig. 4f ranged from 89 to 128 nm. The reason for this decline in the particle size might be attributed to the presence of a large amount of NL in the reaction medium that hindered the propagation step.

### 3.4 Electrical and dielectric properties

**Fig. 5:**
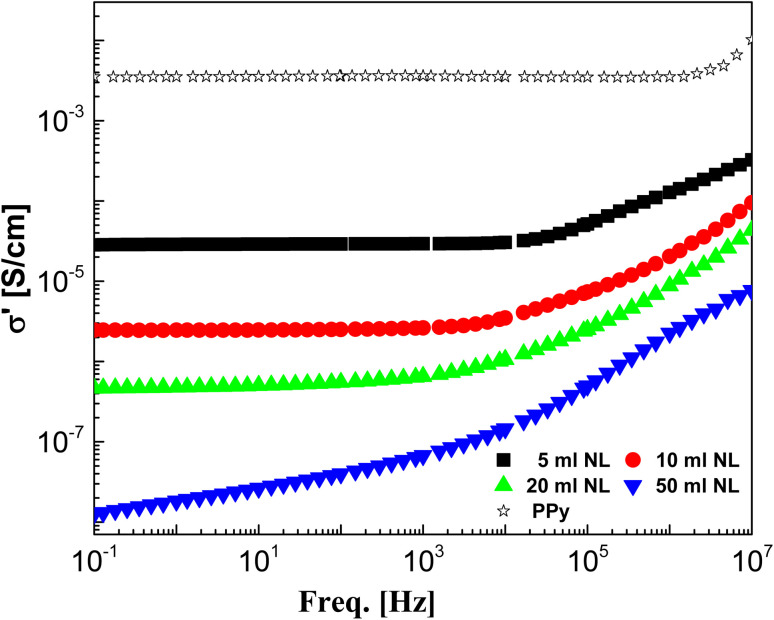
AC-conductivity of polypyrrole/nanolignin composites compared with pure PPy as illustrated graphically against frequency at ambient temperature.

The real part of complex conductivity, *σ*′, was measured for the investigated PPy/NL composites and presented as a function of frequency ranging from 0.1 Hz to 10 MHz in Fig. 5 in comparison with that of the PPy.

**Fig. 6:**
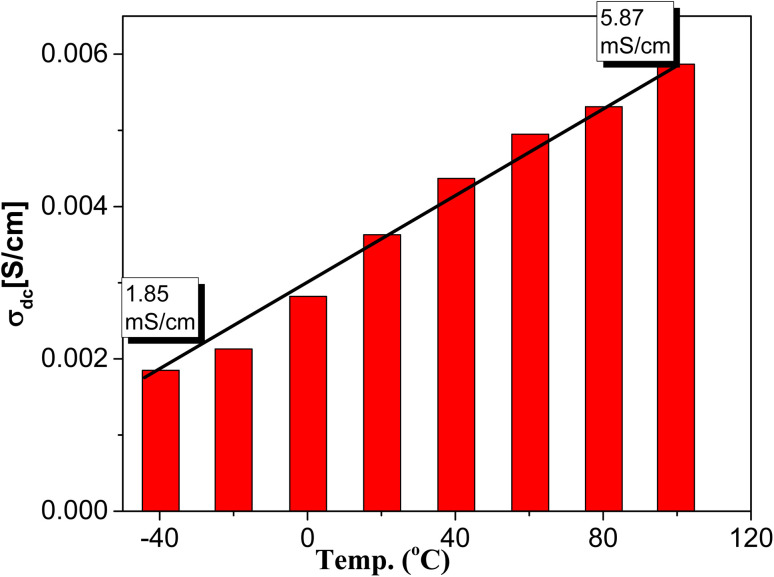
The determined dc-conductivity, *σ*_dc_, of PPy at different temperatures.

**Fig. 7:**
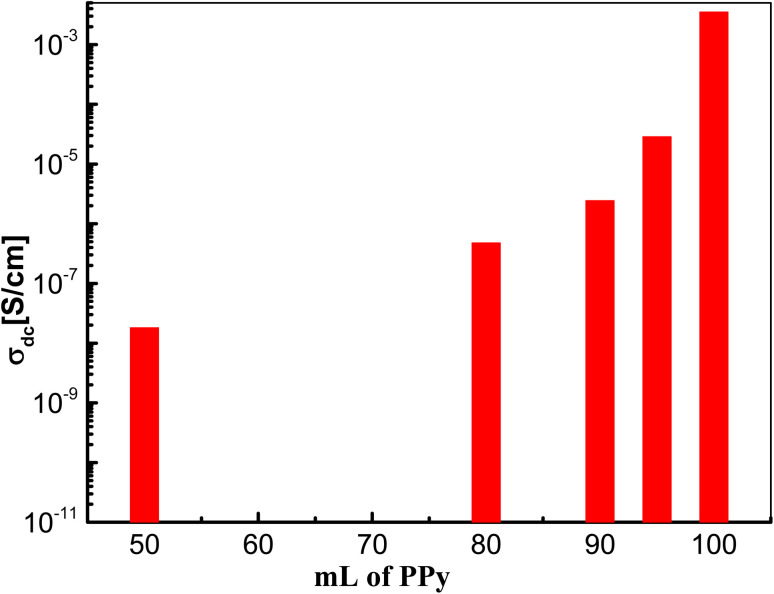
The determined dc-conductivity, *σ*_dc_, at room temperature for different PPy/NL hybrids.

It became a well-known feature in such conducting and semiconducting polymer composites that the frequency behavior of the ac-conductivity at lower and intermediate frequencies shows some case of frequency independence or what is usually called plateau-like behavior that directly yields the dc-conductivity.^53–55^ The determined dc-conductivity, *σ*_dc_, of the pure PPy was illustrated against temperatures varied between −40 and 100 °C in Fig. 6. The figure shows that *σ*_dc_ increases linearly from 1.85 × 10^−3^ S cm^−1^ at −40 °C to 5.87 × 10^−3^ at 100 °C *i.e.* increased only about three times with increasing temperature from −40 to 100 °C. This indicates the slight effect of temperature on the enhancement of the conductivity of PPY. On the other hand, the dc-conductivity of the different concentrations of PPy/NL hybrids as presented in Fig. 7 shows the high effect of NL in reducing the conductivity of PPy. Fig. 5 revealed that the addition of 5% NL decreased dc-conductivity from 3.53 × 10^−3^ of PPy pure to 2.88 × 10^−5^*i.e.* about two orders of magnitude lower. However, increasing NL content to 50% reduces the dc-conductivity to be 1.82 × 10^−8^*i.e.* by about 6 decades.

**Fig. 8:**
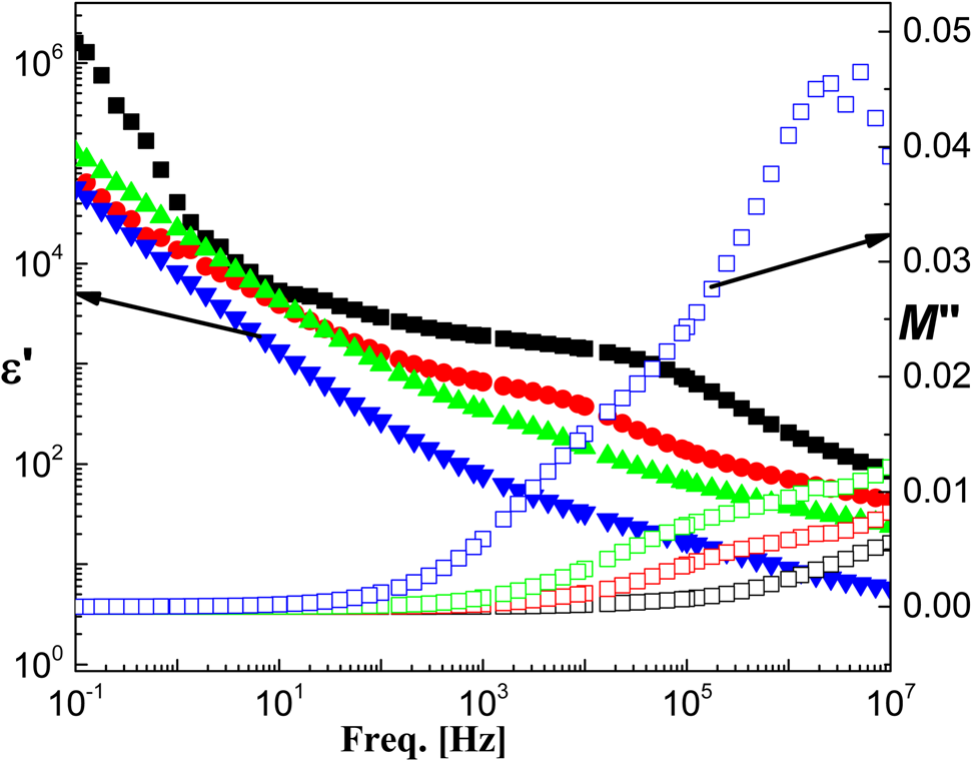
The frequency dependence of permittivity, *ε*′ (closed symbols), and electric modulus loss, *M*′′ (open symbols) of the investigated PPy/NL hybrids. Similar notations as in Fig. 5.

The ac-conductivity (defined as the real part of the complex conductivity), *σ*′, is characterized by the low frequency and intermediate frequencies *via* a plateau that directly yields the dc-conductivity, *σ*_dc_ (Fig. 5). The plateau followed by some characteristic frequency, *ν*_c_, at which dispersion sets in and turns into a power law at higher frequencies. Fig. 8 shows the permittivity, *ε*′ as presented against frequency for the investigated hybrids. At the characteristic frequency (*f*_c_), the permittivity turns from the high frequency limit to the static value, *ε*_s_. Their dispersion step is a direct measure for the dipole moment and hence the polarity of the investigated system. Further decrease of frequency shows a mostly linear increase of the permittivity indicating the contribution of the conductivity. The other side of Fig. 8 shows the electric loss modulus *M*′′ presentation. It shows the contribution of the interfacial polarization or what is usually called Maxwell–Wagner–Sillars (MWS) polarization relaxation that became very clear as the ratio of NL increased.^55–58^ This is the origin of the dispersion step of the relaxation dynamic shown in the permittivity representation.

### 3.5 Optical properties of PPy/NL

**Fig. 9:**
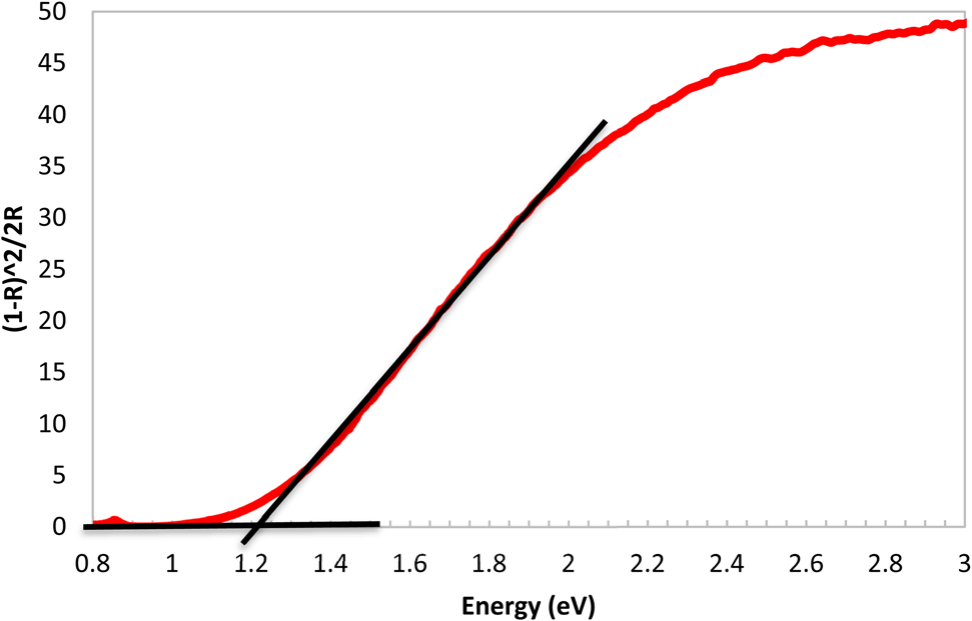
Kubelka–Munk photo absorption graph of NL energy gap in (eV).

The band gap energies of all powder samples were measured using a UV-vis spectrophotometer equipped with a DRA attachment. The band gaps were calculated using the Kubelka–Munk (KM) remission function, which converts the reflectance data to pseudo-absorption, commonly used approach for estimating band gaps in semiconducting materials.^59,60^ The band gap values were determined by extrapolating a least squares linear regression of the initial significant absorption onset until it intersects with the baseline. The pure NL sample exhibits a clear absorption edge with a band gap of 1.25 eV, falling within the near-infrared region (Fig. 9), consistent with its characteristic brown color. In contrast, the KM plots of all NL/PPy composites show a broad, flat absorption response with no distinct onset, indicating a significant quenching of the band gap. This suggests a loss of semiconducting behavior, likely due to electrostatic interactions between NL and PPy, which alter the electronic structure and density of states in the composites.

## 4. Utilization of PPy/NL in DSSC

The PPY electrolyte and its composite with NL at varying concentrations were utilized to fabricate DSSCs (0.5 × 0.5 cm^2^) and tested under simulated sunlight at an incident power of 100 mW cm^−2^. The current–voltage (*I*–*V*) characteristics were recorded, and the fill factor (FF) and efficiencies (*η*) were calculated using eqn (1)–(3) and are presented in Table 1. The NL electrolyte exhibited significantly higher *V*_oc_ and *I*_sc_ compared with the liquid electrolyte, resulting in higher power output (*P*_m_) and efficiency (0.224%). However, the FF is lower, suggesting that while NL can generate more power, it may not be as efficient at converting this power into usable electricity. This improvement in *V*_oc_ is likely due to NL's insulating character and abundant surface functional groups, which may suppress charge recombination at the TiO_2_/dye/electrolyte interface and help maintain a higher quasi-Fermi level in the TiO_2_ conduction band. However, upon increasing the PPy into in the PPy/NL composites (PPy/NL10 to PPy/NL50), a gradual decline in *V*_oc_ was observed. *V*_oc_ values decreased from 497 mV (PPy/NL50) to 364 mV (PPy/NL10), indicating that increasing PPy content leads to higher ionic conductivity but also promotes recombination losses and/or modifies the redox potential of the electrolyte.

**Table 1:** Shows the main influencing factor in the electrolyte composition of the DSSC and the electric factors (*V*_oc_, *I*_sc_, *P*_m_, FF, and eff.) output

Electrolyte	*V*_oc_ (mV)	*I*_sc_ (mA)	*P*_m_ (mW)	FF (%)	Eff. (%)
Liquid (KI/I_2_)	251	0.117	0.018	62.09	0.073
NL	588	0.220	0.056	42.60	0.224
PPy/NL50	497	1.101	0.244	44.50	0.974
PPy/NL20	464	0.111	0.033	64.17	0.133
PPy/NL10	364	0.086	0.020	62.45	0.079
PPy	505	0.914	0.347	75.23	1.389

**Fig. 10:**
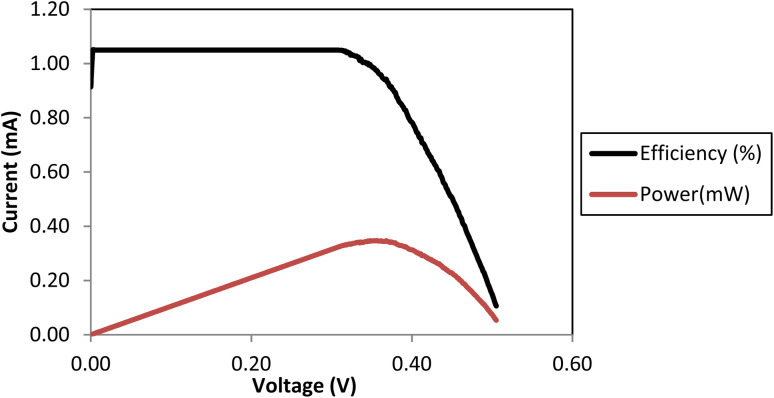
Efficiency (%) and power (mW) of the 0.5 × 0.5 cm^2^ DSSC assembled with PPy electrolyte.

Despite the decrease in *V*_oc_, the overall device efficiency improved with increasing PPy content. The PPy/NL50 composite achieved the best performance among the composites, with an efficiency of ∼1.0%, compared to just 0.2% for pure NL. This enhancement is attributed to improved charge transport and ionic mobility offered by PPy. The FF values (∼60–65%) observed in these composites further confirm stable cell assembly and favorable electrode–electrolyte compatibility. The pure PPy electrolyte (Fig. 10) exhibited the highest efficiency of approximately 1.4%, with a *V*_oc_ of 505 mV and a high FF of ∼75%, owing to its high ionic conductivity and effective redox mediation. In contrast, the low performance of the liquid electrolyte sample, which also showed a low *V*_oc_ and *J*_sc_, suggests that some of the low-current results may also stem from contact resistance or measurement artifacts in the test setup. These factors are being further investigated to refine future measurements. Overall, these results confirm that the electrolyte composition—specifically the PPy/NL ratio—has a direct impact on *V*_oc_ and overall device performance, and that an optimal balance between conductivity and recombination suppression is critical for maximizing DSSC efficiency.

1 *P*_m_ = (*V* × *I*)_max_

2
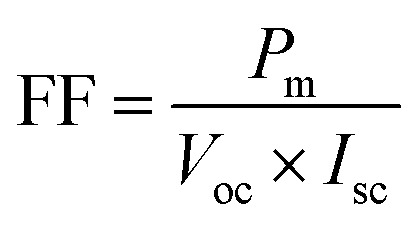
3
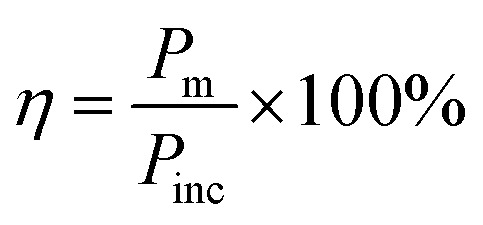
where (FF) is the fill factor, (*P*_m_, *P*_inc_) are the power at maximum power point and incident power; respectively, (*V*_oc_) is the open circuit voltage, (*I*_sc_) is the short circuit current and (*η*) is the efficiency in %.^61^

## 5. Conclusion

In this work nanocomposite based on a biopolymer (NL) and a conducive polymer (PPy) were prepared in different ratios *via in situ* oxidation polymerization. The formed nanocomposite was characterized using FTIR and XRD. Morphology investigations by SEM revealed that the particles have a spherical shape and their size is reduced significantly by increasing the amount of NL in the nanocomposite. The materials showed reduced electrical conductivity with an increasing amount of NL. Nevertheless, investigation of the electrical and dielectric properties of the nanocomposite revealed that an interfacial polarization has occurred originating from the accumulation of charge carriers at the PPy–NL interface. Moreover, NL demonstrated a band gap of 1.25 eV in the near-infrared (IR) region. When used as an electrolyte in dye-sensitized solar cells (DSSCs), the conducting polymer polypyrrole (PPy) significantly improved the performance of NL-based electrolytes. The PPy/NL50 composite showed a notable enhancement in cell efficiency, reaching approximately 1%, compared to only 0.2% for pure NL. This improvement emphasizes the potential of PPy to enhance the performance of NL as an electrolyte in DSSCs. Further optimization of device parameters such as electrolyte thickness, composition, and cell architecture will be essential to fully evaluate and improve the effectiveness of the NL–PPy composite strategy.

## Author contributions

Ebtesam H. A. Eladgham: methodology, investigation, writing – original draft. Mona H. Abdel Rehim: writing – original draft, validation, methodology, investigation, funding acquisition, conceptualization. Gamal M. Turky: methodology, investigation. writing – review & editing, visualization, validation.

## Conflicts of interest

The authors declare that they have no known competing financial interests or personal relationships that could have appeared to influence the work reported in this paper.

## Data Availability

The authors confirm that the data supporting the findings of this study are available within the article.
